# EXPERIENCES OF FAMILY CAREGIVERS OF INDIVIDUALS WITH HYPERTENSION AND DIABETES: A META-ETHNOGRAPHY

**DOI:** 10.1093/geroni/igae098.3969

**Published:** 2024-12-31

**Authors:** Ashwag Alhabodal, Confidence Francis- Edoziuno, Marsha Williamson, Wendy Henderson, Ruth Lucas

**Affiliations:** University of Connecticut, Storrs, Connecticut, United States; University of Connecticut, Storrs, Connecticut, United States; Pace University, New York, New York, United States; University of Pennsylvania, Philadelphia, Pennsylvania, United States; University of Connecticut, Storrs, Connecticut, United States

## Abstract

The number of older adults diagnosed with chronic diseases such as hypertension or diabetes mellitus is growing, and their home care is often managed by family caregivers. The purpose of this meta-ethnography is to synthesize qualitative evidence, published between 2014 and 2024, and identify common challenges, motivations, and coping strategies of caregivers. Ten articles were included, conducted in diverse settings across high-, middle-, and low-income countries with 261 caregivers, mostly females. Through Noblit and Hare’s approach, five overarching themes were identified: caregiving expertise and knowledge, psychosocial impact, support and coping mechanisms, financial and physical burdens, and intrinsic motivations and values. Many caregivers receive inadequate formal training and resources, which often lead to intense emotional, financial, and physical burdens. These personal burdens were balanced by their strong personal values and motivation to provide care. The voices of caregivers reveal not only the depth of their commitment but also the significant cost caregiving takes on their mental and emotional health. Caregivers speak of isolation and anxiety, as well as the resilience they draw from family bonds and religious beliefs. Their narratives highlight the universal challenges experienced by caregivers globally. However, the availability and quality of support systems may differ widely. Caregivers have a critical role in the management of chronic diseases for their older adult family members, and they require interventions and policy changes to ensure their well-being and the quality of care they provide.

